# Health Information Technology in Healthcare Quality and Patient Safety: Literature Review

**DOI:** 10.2196/10264

**Published:** 2018-06-04

**Authors:** Sue S Feldman, Scott Buchalter, Leslie W Hayes

**Affiliations:** ^1^ Department of Health Services Administration The University of Alabama at Birmingham Birmingham, AL United States; ^2^ Pulmonary and Critical Care The University of Alabama at Birmingham Medical Center Birmingham, AL United States; ^3^ Department of Pediatrics The University of Alabama at Birmingham Medical Center Birmingham, AL United States

**Keywords:** Health Information Technology, Healthcare Quality, Patient Safety

## Abstract

**Background:**

The area of healthcare quality and patient safety is starting to use health information technology to prevent reportable events, identify them before they become issues, and act on events that are thought to be unavoidable. As healthcare organizations begin to explore the use of health information technology in this realm, it is often unclear where fiscal and human efforts should be focused.

**Objective:**

The purpose of this study was to provide a foundation for understanding where to focus health information technology fiscal and human resources as well as expectations for the use of health information technology in healthcare quality and patient safety.

**Methods:**

A literature review was conducted to identify peer-reviewed publications reporting on the actual use of health information technology in healthcare quality and patient safety. Inductive thematic analysis with open coding was used to categorize a total of 41 studies. Three pre-set categories were used: prevention, identification, and action. Three additional categories were formed through coding: challenges, outcomes, and location.

**Results:**

This study identifies five main categories across seven study settings. A majority of the studies used health IT for identification and prevention of healthcare quality and patient safety issues. In this realm, alerts, clinical decision support, and customized health IT solutions were most often implemented. Implementation, interface design, and culture were most often noted as challenges.

**Conclusions:**

This study provides valuable information as organizations determine where they stand to get the most “bang for their buck” relative to health IT for quality and patient safety. Knowing what implementations are being effectivity used by other organizations helps with fiscal and human resource planning as well as managing expectations relative to cost, scope, and outcomes. The findings from this scan of the literature suggest that having organizational champion leaders that can shepherd implementation, impact culture, and bridge knowledge with developers would be a valuable resource allocation to consider.

## Introduction

### Background

It has long been known and accepted that healthcare in the US is too expensive and the outcomes are less than predictable [1]. The turn of the century brought with it a realization that healthcare, like other industries, could use data to increase our awareness of seemingly uncontrollable costs and unpredictable outcomes. With almost two decades of compiling, analyzing, mashing up data, and trying to make sense of how the data inform multiple layers of healthcare, it is time to look beyond the awareness that the data provide, and instead develop an understanding of how to use the data for predictable and actionable purposes, especially with regard to healthcare quality and patient safety. The literature is mixed on the degree to which health information technology (IT) as a valuable suite of tools, applications, and systems that have contributed to actual savings and efficiencies [1-4]. However, the area of healthcare quality and patient safety lends itself to many of the same business intelligence and predictability advantages that are seen in the credit card industry [5-7].

Much like the Triple Aim of Healthcare, the credit card industry is working toward decreased costs (fraud), increased quality (better transactions), and increased satisfaction (happier merchants and happier cardholders). The credit card industry began using business intelligence to predict behavior that suggested fraud, developed process maps for transaction processing, and offered perks to merchants and cardholders. Just as the credit card industry learned from healthcare, healthcare can borrow from the credit card industry to use healthcare intelligence for prevention, identification, and action related to healthcare quality and patient safety events.

**Figure 1:**
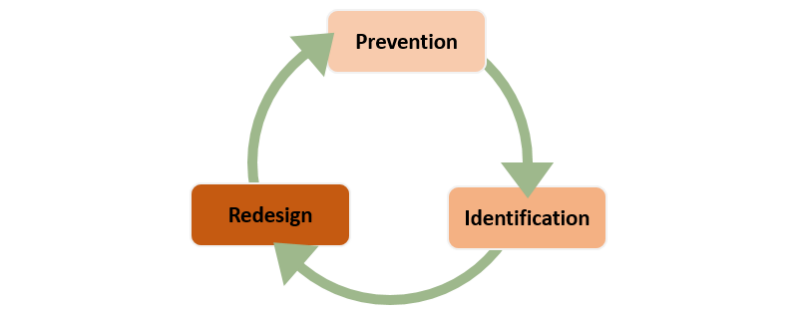
Improving the reliability of healthcare.

**Figure 2:**
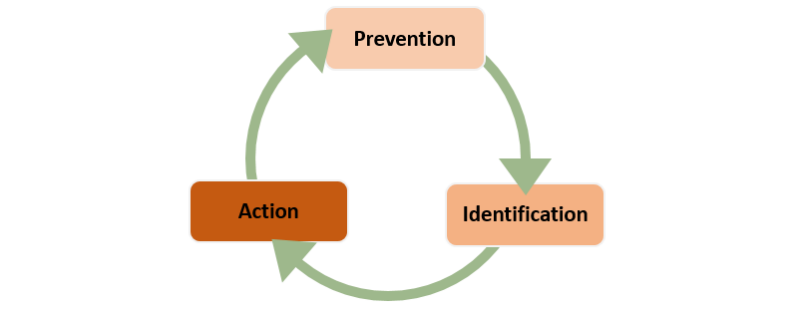
Improving the reliability of healthcare quality and patient safety.

The Institute for Healthcare Improvement (IHI) suggests that reliability around healthcare is a three-part cycle of failure prevention, failure identification, and process redesign and defines reliability as “failure-free operation over time.” [8]. Other areas of healthcare have used information systems to provide continuous monitoring with real-time, or near real-time reporting as a means of achieving reliability [9]. As such, it makes sense to think about the role of health IT in reliability as it relates to healthcare quality and patient safety. A review of the literature suggests that healthcare organizations are using health IT for healthcare quality and patient safety and that they have replaced *redesign* in Figure 1 with *action* as shown in Figure 2 [10-12]. Action, in this case, allows for health IT to be implemented after a potential healthcare quality or patient safety event has occurred and does not necessarily require a redesign. Ordering alerts in the electronic health record are an example of action; the event has occurred (the order has been entered) and health IT in the form of an alert is initiated to stop the potentially unsafe order from being filled by the pharmacy.

Having an understanding of this cycle helps to create awareness around where various applications of health IT find their “best fit” in improving the reliability of healthcare quality and patient safety. A distinct advantage of this being a “cycle” is that there is no defined beginning and ending point, but rather an insertion point. This is all to say that the cycle should not be interpreted as starting with prevention and ending with action.

### Health Information Technology for Prevention of Quality and Safety Events

Health IT for prevention of quality and safety events involves the use of health IT to *prevent a quality and safety event from even happening*. Automated reminders and alerts are useful in providing essential information that supports safe and effective clinical decisions [13]. Such alerts in the electronic health record (EHR) are a standard mechanism for the use of health IT for prevention of potential missed quality and patient safety events. For example, immunization alerts have led to a 12% increase in well-child and a 22% increase in sick child immunization administration [14] and drug alerts have been associated with a 22% decrease in medication prescription errors [15]. Soft-stops can provide key information about a potential quality or patient safety issue. They may offer choices but usually, require only that the user acknowledge the alert to proceed.

A hard-stop, on the other hand, prevents the user from moving forward with an order or intervention that would be potentially dangerous to a patient. Hard-stops may allow continuation of the process, but only if significant required action is taken by the user, such as a call to or consultation with an expert (such as a pharmacist or a medical specialist). In some cases, soft-stops might be ignored or overridden because of such issues as alert fatigue, poor implementation, or poor interface design [16,17]. Hard-stops, when appropriately designed, have been shown to be more successful in changing an unsafe plan or preventing a potentially dangerous intervention [18,19].

### Health Information Technology for Identification of Quality and Safety Events

Health IT for identification of quality and safety events involves health IT that is used to *identify a quality and safety event when it is about to occur*. Health insurance providers increasingly place pressure on healthcare systems to reduce the cost of care delivery and improve patient outcomes. This pressure may exist through tiered reimbursement structures, benefitting those systems which meet or exceed specific benchmarks of performance. Growing pressure from these payers takes the form of non-reimbursement for care determined by the payer to be unnecessary or in excess of “standard care.” Health IT can be used to find the EHR populations of patients for whom reimbursement might be lower than expected. One such example to consider is the length of stay for a particular procedure. While the use of health IT can produce reports and dashboards that are helpful for decision-making relative to reimbursement trends and practices for lengths of stay for that diagnosis, it is crucial that thoughtful consideration be given for appreciating any unintended consequences. For example, when reducing the length of stay, unintended readmissions are an important metric to follow.

### Health Information Technology for Action in Quality and Safety Events

Health IT for action of quality and safety events involves health IT that is used to a*ct on a quality and safety event once it has already occurred.* That is to say that these are actions that were reported in the literature that were taken *as a result of* an event. Health IT for action differs from health IT for prevention in that the former is a reaction directly correlated to an event reported in the article, whereas the latter is reported in the article as a preemptive measure, in advance of an event.

Because of their standardization, there are several clinical care pathways that lend themselves to clinical decision support. One such clinical care pathway is sepsis. Despite nearly two decades of advances in early sepsis care, sepsis outcomes persist to be poor, and sepsis remains a leading cause of death worldwide and accounts for significant morbidity and mortality [20]. In light of this, there is a growing national push to increase early identification and treatment of sepsis with a goal of improving outcomes. Patients with sepsis are some of the most critically ill patients admitted to hospitals, and survival depends heavily upon timely and early administration of key interventions followed quickly by assessing and acting on results of these interventions [21]. Some examples include administration of IV antibiotics and aggressive IV fluids within one hour [21]. Examples of assessments of interventions include measuring specific physical and laboratory values that provide crucial information about the patient response. All too often, clinicians are faced with an overabundance of data, that while all necessary, may not be relevant to the issue at hand. For example, lab results might be presented in their entirety, when in practice, there are only 3 or 4 tests that will drive decision-making. The difficulty is how to separate the noise (non-essential at that moment) from the signal (essential at that moment). Health IT solutions, such as dashboards and other solutions can be used to ensure that essential data are in a primary viewing position and non-essential data in a secondary viewing position (perhaps on drill down, for example).

This paper will provide foundational knowledge and understanding for organizations of where to focus health IT fiscal and human resources. It will also provide information relative to some of the challenges that can be expected in implementing health IT for quality and patient safety.

## Methods

**Figure 3:**
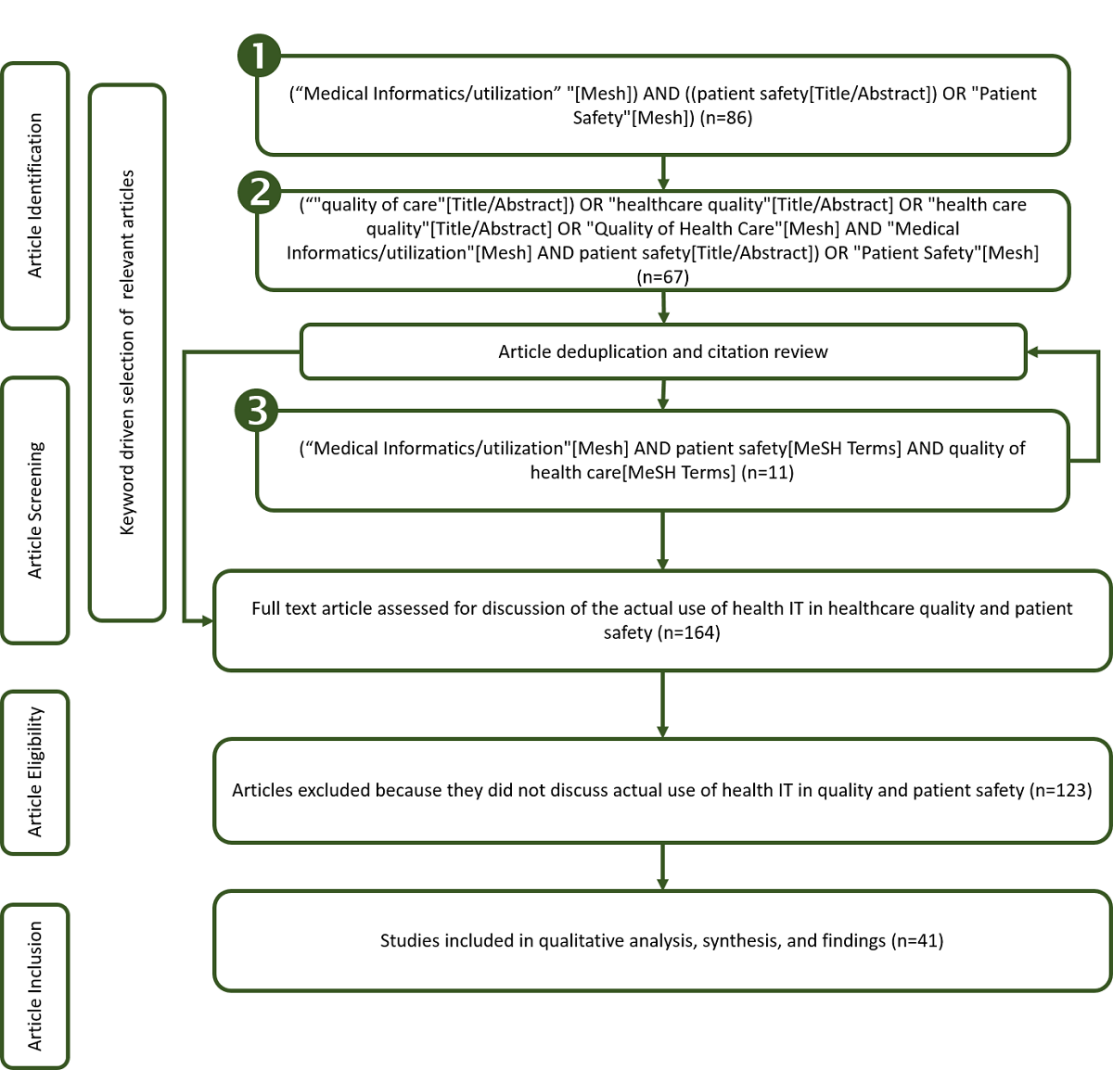
Literature search process.

This review of the literature took a structured approach using PubMed and a combination of keywords. Since PubMed indexes peer-reviewed articles from biomedical information, it was felt that this was the most appropriate and inclusive source. A healthcare-focused librarian, under the direction of all authors, conducted the literature search. The articles for final selection were discussed and decided upon among the authors. The structured approach was guided by the model illustrated in Figure 3.

The process to article inclusion involved three passes to collect publications related to health IT in quality and patient safety for peer-reviewed studies published between 2012-2017, inclusive. The first pass, (shown as “1” in Figure 3), used high-level keywords and returned 86 full-text articles. From the articles gathered, additional keywords were added to the search. After deduplication and citation review, the second pass (shown as “2” in Figure 3) added 67 unique full-text articles. After deduplication and citation review, the third pass (shown as “3” in Figure 3) added 11 unique full-text articles, for a total of 164 unique full text articles. Each article was further analyzed to identify the degree to which the article discussed health IT in healthcare quality and patient safety. To be considered for inclusion, the study needed to report on the *actual* use of health IT in healthcare quality and patient safety. Forty-one studies met these criteria. Those studies with their contributions to the results are shown in the results section of this paper.

Qualitative data analysis software (Atlas.ti 8 for Windows) was used in directed content analysis as a method to categorize and code the 41 studies relative to *how* health IT was used in healthcare quality and patient safety. All 41 documents were uploaded into the document manager in Atlas.ti as Primary Documents (PD). During this process, the article title was used as the PD name. Inductive thematic analysis with open coding was used under the three pre-set categories of prevention, identification, and action [22]. This allowed for capturing descriptions of how health IT was used in each circumstance.

For example, prevention included descriptions of any use of health IT to prevent quality issues or potential safety events, identification included any descriptions of the use of health IT to identify quality issues or safety events, and action included any descriptions of the use of health IT to act on quality issues or safety events that have occurred. When content was noted that did not fit into the three pre-set categories, an additional category was created. Additional categories were created to capture challenges relative to the use of health IT in quality and patient safety. Since some papers discussed how the use of health IT impacted health outcomes, an additional category was created for outcomes. Lastly, an additional category was created to capture the study settings or location.

The coding structure was agreed upon by all authors, and one author conducted the coding. After all of the studies were coded, two additional passes were made through the data. The first pass was to ensure that all information from the studies that should be coded was actually coded and coded to the correct code (ie, was a passage that described prevention actually coded to prevention?). The second pass was to consider sub-categories for consolidation. Six sub-categories were consolidated.

The purpose of examining co-occurrences is to understand what, if any, relation exists between concepts [22,23]. Within Atlas.ti, a co-occurrence table was run to find codes that co-occur across the literature, the purpose of which was to illuminate the areas most discussed. This table was then exported to Microsoft Excel for further analysis.

Network maps are a means by which analysis can be visualized in relationships to provide a different perspective on the codes, categories, etc., and with that visualization, provide a mechanism for moving codes around [22]. Those presented in the results do not differ from the final coding structure, but instead are used to provide a visual representation.

## Results

### Overview

Literature reviews can be conducted using a qualitative approach [24,25] with the results displayed in a variety of ways to support models and show connections [22]. As such, this review presents qualitative findings to support the “improving the reliability of healthcare quality and patient safety” model introduced earlier in this paper and shows connections via network mappings in Figure 6 through Figure 7 and co-occurrences in Table 2.

**Figure 4:**
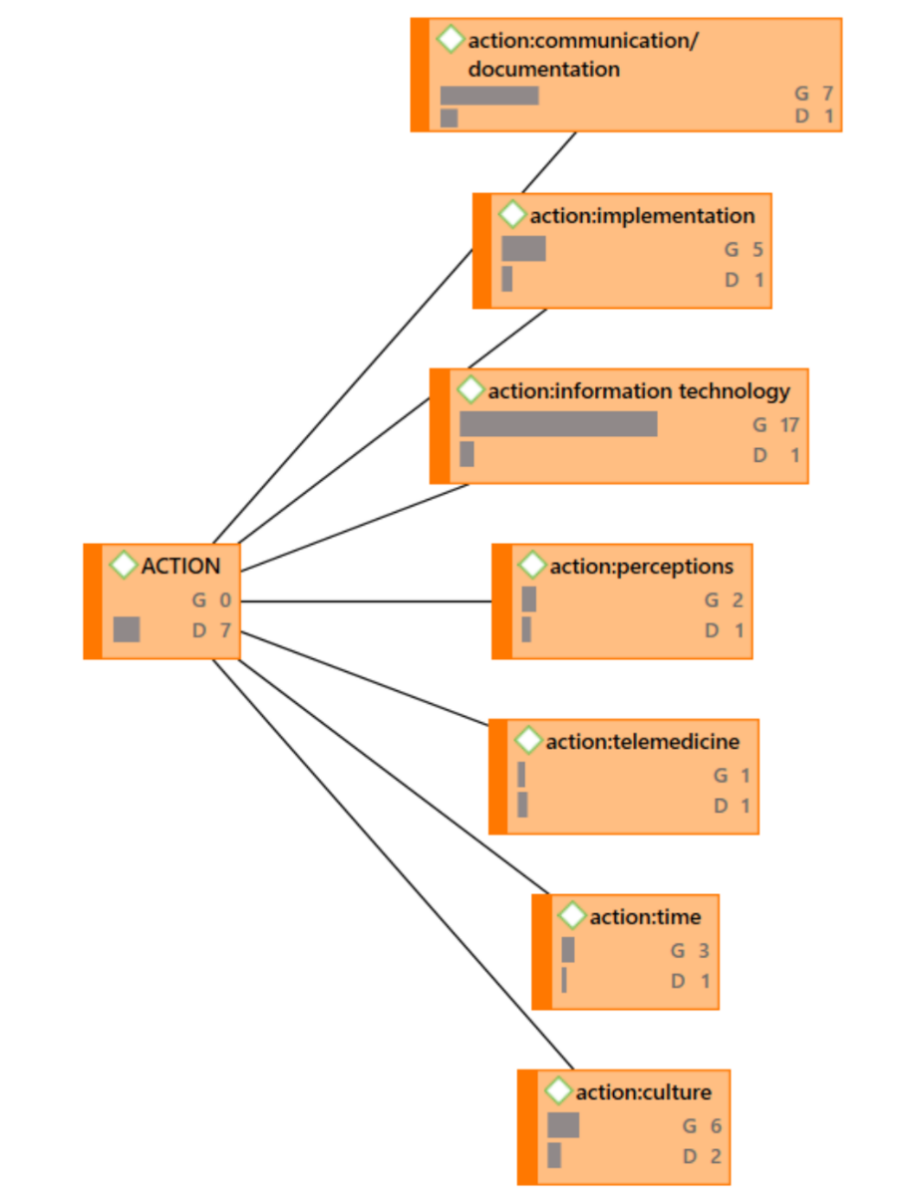
ACTION Network Diagram (G=groundedness, D=density).

Table 1 provides a listing of the articles and their contribution in this results section to support the model (Figure 2), network maps (Figure 4 through Figure 7), and co-occurrences (Table 2).

From the 41 studies that fit the inclusion criteria, any element in which the authors discussed the use of health IT for healthcare quality and patient safety was identified, even if it did not fit into the three previously determined categories. This process yielded a total of 50 codes across five categories: action (7/41, 17.1%), challenges (12/41, 29.3%), identification (10/41, 24.4%), outcomes (5/41, 12.2%), and prevention (16/41, 39.0%) across seven study settings. Just under a quarter of the studies identified a study setting: anesthesia (2/41, 4.9%), behavioral health (1/41, 2.4%), emergency department (2/41, 4.9%), any intensive care unit (3/41, 7.3%), clinical diagnostic laboratory (1/41, 2.4%), pediatrics (2/41, 4.9), surgery (1/41, 2.4%).

Across all of the articles, there were 63 and 92 descriptions of the use of health IT for identification and prevention of healthcare quality and patient safety issues, respectively. Health IT for action and the challenges associated with health IT for healthcare quality and patient safety was described 41 and 43 times, respectively.

The findings from the literature review are presented by the categories outlined in the previously introduced model for improving the reliability of healthcare quality and patient safety.

### Prevention

The first exploration was across the literature that discussed health IT for *prevention* of quality and patient safety issues to see exactly how organizations were reporting health IT use to *prevent a quality and safety event from even happening*. The greatest areas of use were around alerts [30,31,44,56,58], clinical decision support [39,44,47,56], implementation [10,32,37,38,56], interface design [26,34,42,45,56,59], and customized health IT solutions [29,30,32,34,46-50,56,58,59]. Customized health IT solutions were anything that described the use of health IT but lacked any specificity beyond that described in this section. For example, this could be something as simple as checklists or as complex as algorithmic diagnostic trees. To clarify, alerts are a subset of clinical decision support. Since so many of the occurrences specified alerts and clinical decision support separately, these were coded separately. Clinical decision support, by definition, includes alerts, clinical care guidelines, condition-specific orders sets, clinical reports and/or summaries, documentation templates, diagnostic support, and clinical reference support. Implementation and interface design were each described in terms of having been poorly implemented or poorly designed and having implications on utility in healthcare quality and safety.

### Identification

The next exploration was across the literature that discussed health IT for *identification* of quality and patient safety issues; in other words, how health IT was used to *identify a quality and safety event when it is about to occur*. In this regard, similar to prevention (but described differently in the included studies), alerts [26,30,31,44,56,58], clinical decision support [30,31,39,44,56,58], implementation [10,32,38,56], and customized health IT solutions [10,30,31,34,46-49,52,56,58] were most prominent. For example, alerts, clinical decision support, and customized health IT solutions were all described in the literature as having been implemented to identify a potential quality or patient safety issue, yet the literature also described how the implementation of these could have been better in terms of providing more training to those on the receiving end of the alerts, clinical decision support, or other customized health IT solutions.

**Table 1:** Article contribution to results (in alphabetical order). An “X” indicates the area of the results contribution and “—” indicates no contribution.

Citation	Action	Challenges	Identification	Outcomes	Prevention
Ancker et al [10]	X	X	X	—	X
Arabi et al [26]	—	—	X	X	X
Asch et al [27]	—	—	—	—	X
Badrick et al [28]	—	—	—	—	X
Coiera et al [29]	—	X	—	—	X
Colicchio et al [11]	X	—	—	—	—
El Morr et al [30]	—	—	X	—	X
Every et al [31]	—	—	X	—	X
Farzandipour et al [32]	—	X	X	—	X
Gupta and Kaplan [12]	X	—	—	—	—
Hoonakker et al [33]	—	—	X	—	X
Jensen [34]	X	X	X	—	X
Khullar et al [35]	X	—	—	—	—
Kim et al [36]	—	X	—	—	X
Koppel [37]	X	X	—	—	X
Lassere et al [38]	X	X	X	—	X
Levesque et al [39]	X	—	X	X	X
Magrabi et al [40]	X	—	—	—	—
Martin et al [41]	—	X	—	—	—
Mazur et al [42]	X	—	—	—	X
Nakhleh [43]	-—	—	—	—	X
Peters [44]	X	X	X	—	X
Popovici [45]	—	X	—	—	X
Rizzato et al [46]	X	X	X	—	X
Seblega et al [47]	X	—	X	X	X
Shy et al [48]	X	—	X	—	X
Skyttberg et al [49]	—	X	X	X	X
Stanton [50]	X	—	—	—	X
Strickland [51]	—	—	—	—	X
Suresh [52]	—	X	X	—	X
Wang et al [53]	—	X	—	—	X
Weiner [54]	—	—	—	—	X
Whipple et al [55]	X	—	—	—	—
Whitt et al [56]	X	X	X	—	X
Yermak, et al [57]	—	—	—	—	X
Yu et al [58]	—	X	X	—	X

**Figure 5:**
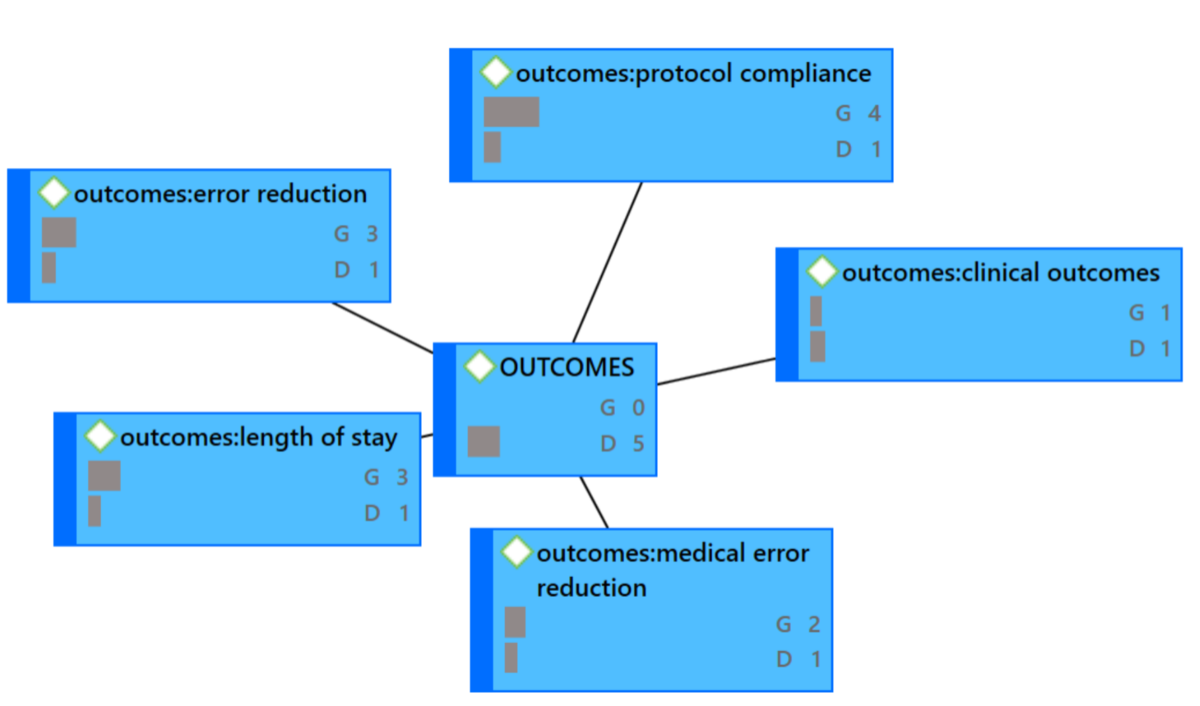
OUTCOMES Network Diagram (G=groundedness, D=density).

**Figure 6:**
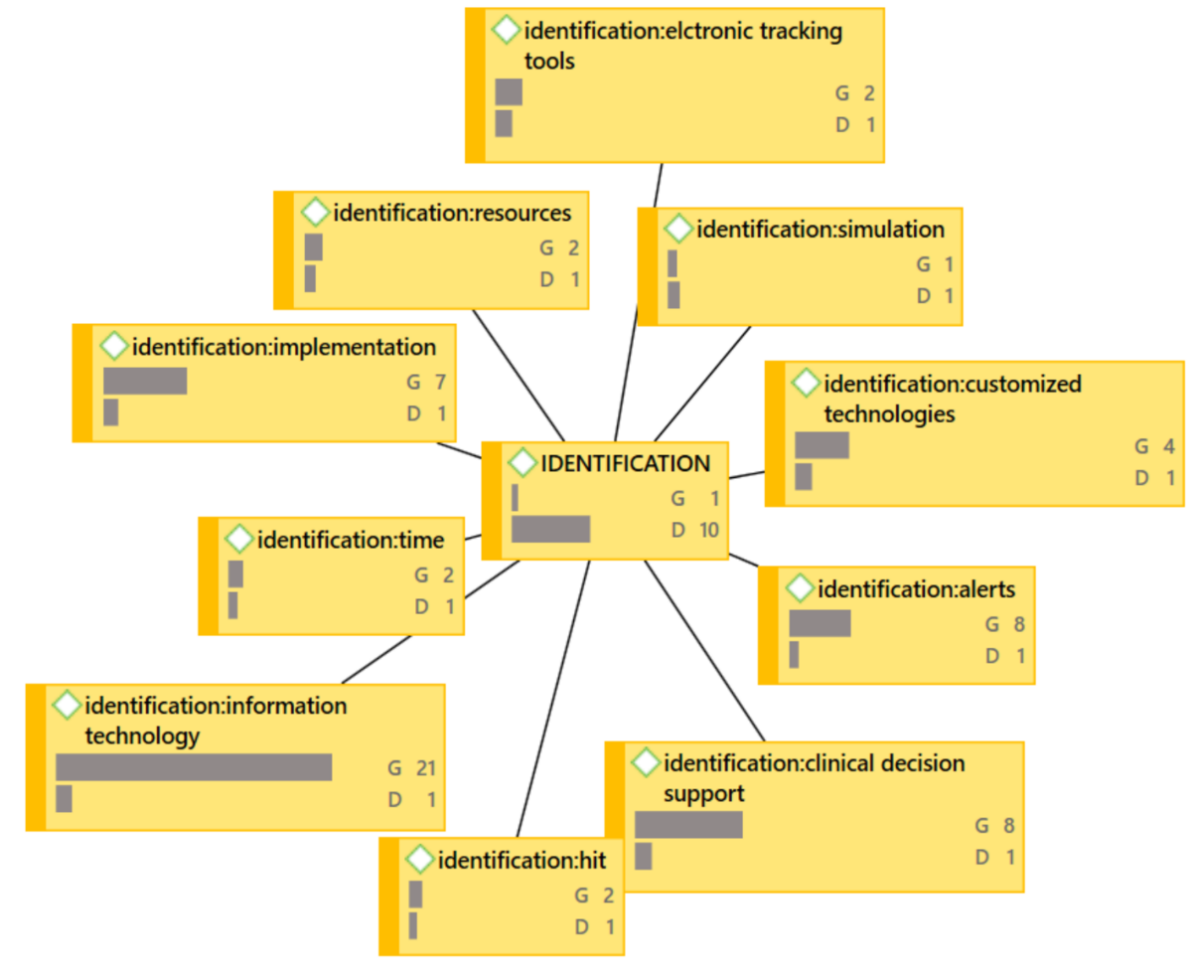
IDENTIFICATION Network Diagram (G=groundedness, D=density).

**Figure 7:**
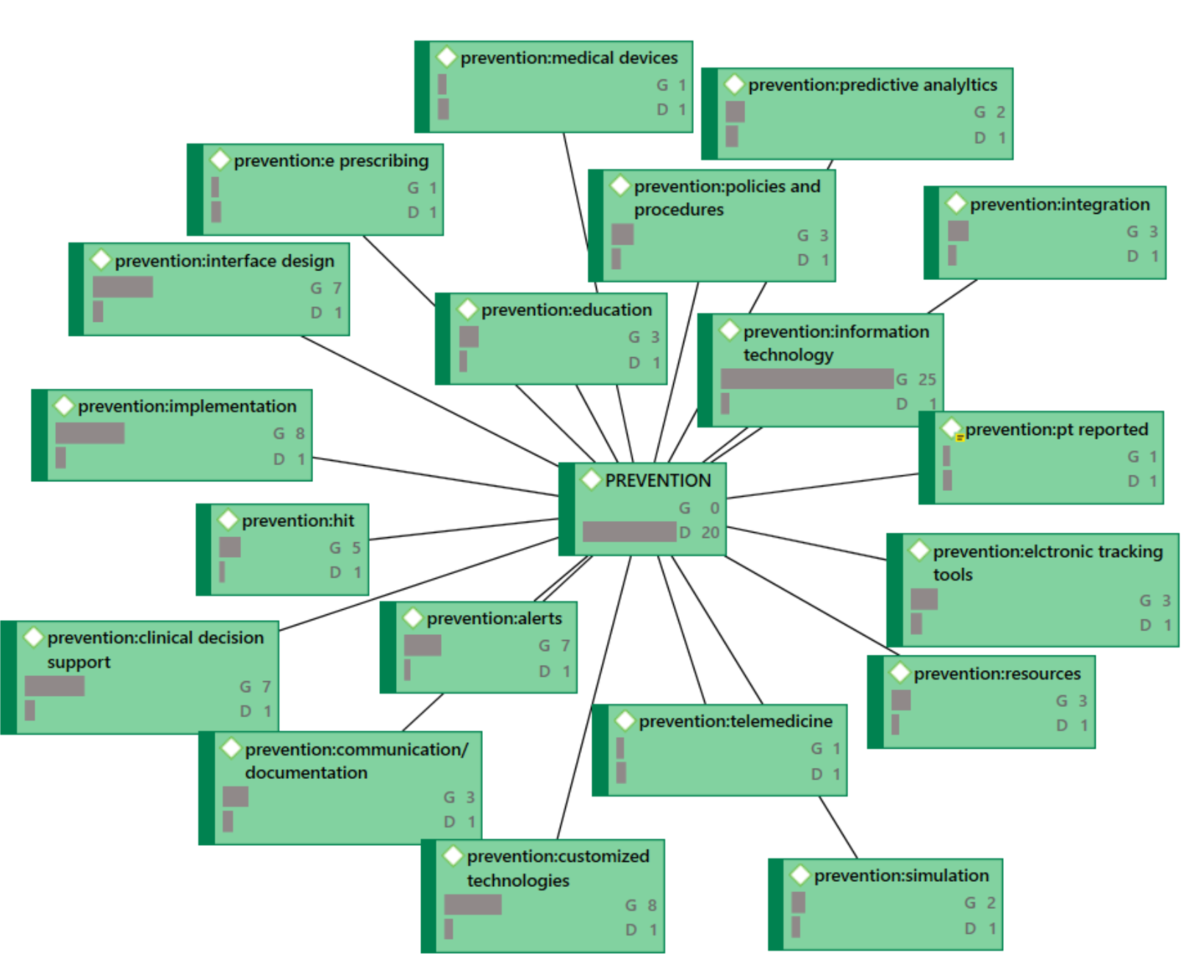
PREVENTION Network Diagram (G=groundedness, D=density).

**Table:** 

Code	Co-occurrences
Implementation	Prevention, Identification, Action, Challenges
Alerts	Prevention, Identification
Clinical decision support	Prevention, Identification
Interface design	Prevention, Challenges
Culture	Action, Challenges (tattling)
Customized health IT^a^ solutions	Prevention, Identification

^a^IT: information technology.

### Action

The third exploration was across the literature that discussed health IT for *action on a quality and safety event once it has already occurred.* That is to say that these are actions that were reported in the literature that were taken *as a result of* an event. In regards to action, the major areas were documentation [10,32,37,41,46,56,58], implementation [10,32,37,58], and culture [10,29,41,53,58] relative to the use of health IT.

The findings from the review of the literature show that implementation appeared in prevention, identification, *and* action. Implementation in general has been demonstrated in the literature as a challenge, and that was revealed in this literature review also. Culture was most often referred to as needing to create a culture of quality and patient safety in order for health IT to be embraced. Organizations that started working on culture change before implementation of health IT solutions suggested that health IT for acting on quality and patient safety events was more favorable. Therefore, the analysis was run with challenges which suggests the major areas are: culture, implementation, and interface design.

### Co-occurrences

Employing the Improving the reliability of healthcare quality and patient safety model introduced in Figure 2 and adding challenges, six critical co-occurrences emerged (see Table 2).

As described earlier, co-occurrences expose relationships exists between concepts [22,23]. The top co-occurring codes in Table 2 create a macro level view of how health IT was most commonly used for quality and patient safety relative to the “improving the reliability of healthcare quality and patient safety” model introduced in Figure 2. However, it is also important to understand the universe of ways in which organizations used health IT for quality and patient safety; in other words, the art of the possible when using health IT for quality and patient safety. Network maps provide a mechanism by which to visualize the connectedness of all data coded across all 41 articles included in this analysis. These maps, along with some quantitative information increase understanding at this universe level (macro and micro views).

In the network diagrams that follow (which also represent the coded categories and sub-categories), G signifies the level of groundedness of the particular code. Groundedness, in this case, indicates the frequency of the code relative to the code category. D signifies the level of density or connectedness of the particular code. Density, in this case, indicates the number of other codes to which this code is connected. For example, under ACTION, Figure 4, the code action: culture shows G6, D2. ACTION is the code category and action: culture is the code “culture” under the ACTION code category (this coding structure helps to maintain alpha order). This can be read as the following: “Culture was described six times across all 41 papers relative to ACTION and is connected to two code categories total.” Because it would make the network diagrams unwieldy, not shown in the exhibits is the specificity around the groundedness or the density. See Figures 4 through Figure 7.

## Discussion

### Principal Findings

This scan of the literature is intended to inform practice. The information from this study could be useful as organizations determine where they stand to get the most “bang for their buck” relative to health IT for quality and patient safety. Centered around the Improving the Reliability of Healthcare Quality and Safety model introduced in Figure 2 and the macro level uses of health IT for quality and patient safety outlined in Table 2, organizations in the planning stages may want to begin with alerts and clinical decision support, understanding that alerts are a subset of clinical decision support. This information also helps with resource planning. For example, implementation appeared in all three categories of the Improving the Reliability of Healthcare Quality and Safety model. Additionally, culture was shown to be a challenge. Organizational leaders know that changing culture can be a long and intensive process. The findings from this scan of the literature suggest that having organizational champion leaders that can shepherd implementation, impact culture, and bridge knowledge with developers would be a valuable resource allocation to consider.

Health IT must meet quality improvement at the intersection with care delivery. From a clinical perspective, this is experienced on several levels, and the solution depends, in part, on the clinical problem to be addressed. Some typical examples of health IT interventions illuminated in the findings include: (1) reminders and alerts, (2) decision support tools, (3) checklists (including order sets and protocols), and (4) soft- and hard-stops.

As noted, this scan of the literature is provided as a means to inform practice. It does not consider further model modification, and this represents an area of future research in the application of health IT for quality and patient safety.

### Limitations

This study is limited in that it used PubMed as a single source for the searching and one coder coded all studies. A more comprehensive and systematic review would include multiple databases and multiple coders. Although all authors reviewed the codes, multiple coders would ensure intercoder reliability, which cannot be assured in this study. Additionally, since all studies reviewed did not include locations, generalizability to all areas of clinical care cannot be certain.

### Conclusion

A review of the literature for this study concluded that organizations in the planning stages of using health IT to improve quality and safety may want to begin with reminders and alerts, decision support tools, and checklists.

## Abbreviations

IT: information technology
IHI: Institute for Healthcare Improvement
EHR: Electronic Health Records
PD: Primary Documents
