# Figure Correction: Health Information Technology in Healthcare Quality and Patient Safety: Literature Review

**DOI:** 10.2196/11320

**Published:** 2019-01-03

**Authors:** Sue S Feldman, Scott Buchalter, Leslie W Hayes

**Affiliations:** 1 Department of Health Services Administration The University of Alabama at Birmingham Birmingham, AL United States; 2 Pulmonary and Critical Care The University of Alabama at Birmingham Medical Center Birmingham, AL United States; 3 Department of Pediatrics The University of Alabama at Birmingham Medical Center Birmingham, AL United States

The authors of “Health Information Technology in Healthcare Quality and Patient Safety: Literature Review” (JMIR Med Inform 2018;6(2):e10264) mistakenly provided revised figures that were not identical in every way to the original ones in the paper that they were meant to replace.

During proofreading, the authors were asked to provide updated versions of Figures 4-7 because the versions that were originally provided were not of sufficient resolution. When creating better quality images, the authors accidentally omitted frequencies (groundedness and densities) that were included in the original images.

The incomplete versions of Figures 4-7 have been replaced and can be viewed below. 

The correction will appear in the online version of the paper on the JMIR website on January 3, 2019, together with the publication of this correction notice. Because this was made after submission to PubMed, PubMed Central, and other full-text repositories, the corrected article also has been resubmitted to those repositories.

**Figure 4:**
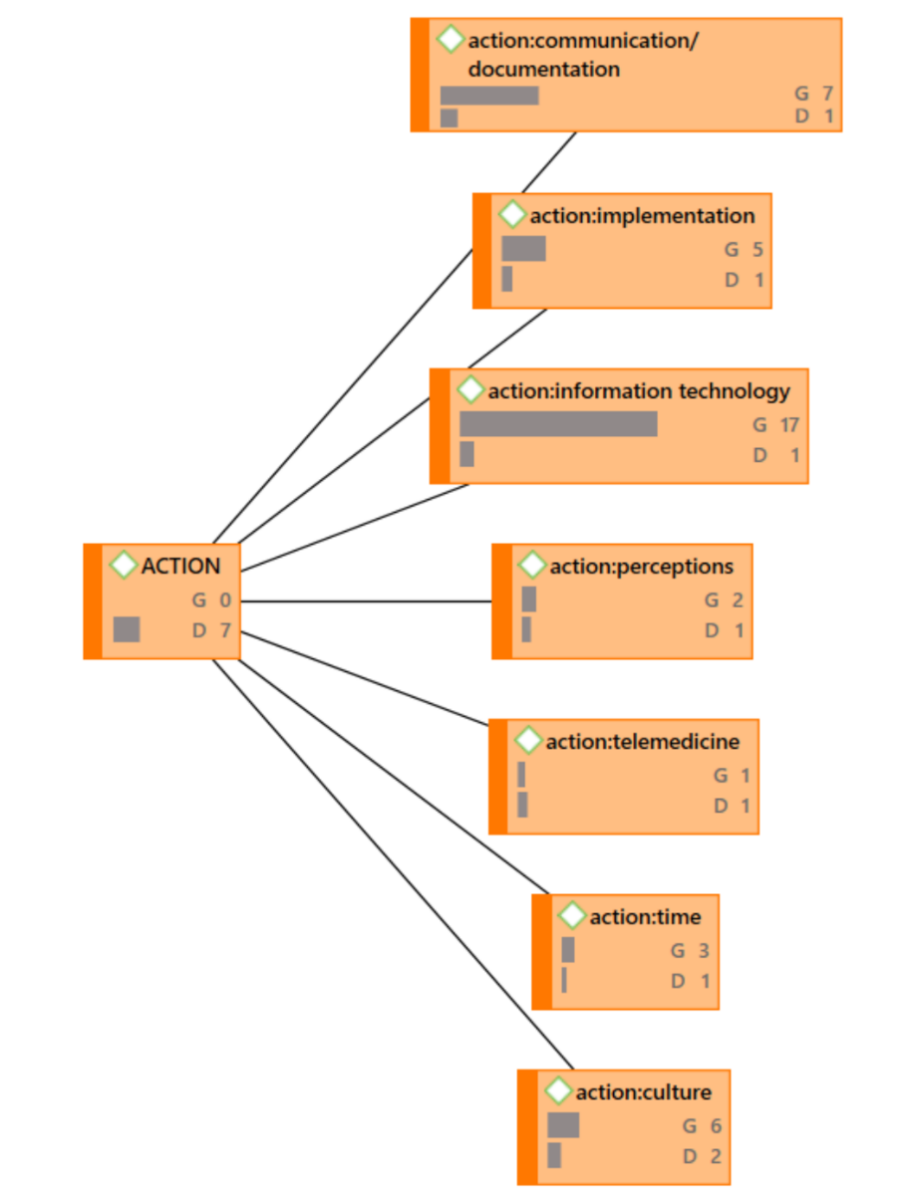
ACTION Network Diagram (G=groundedness, D=density).

**Figure 5:**
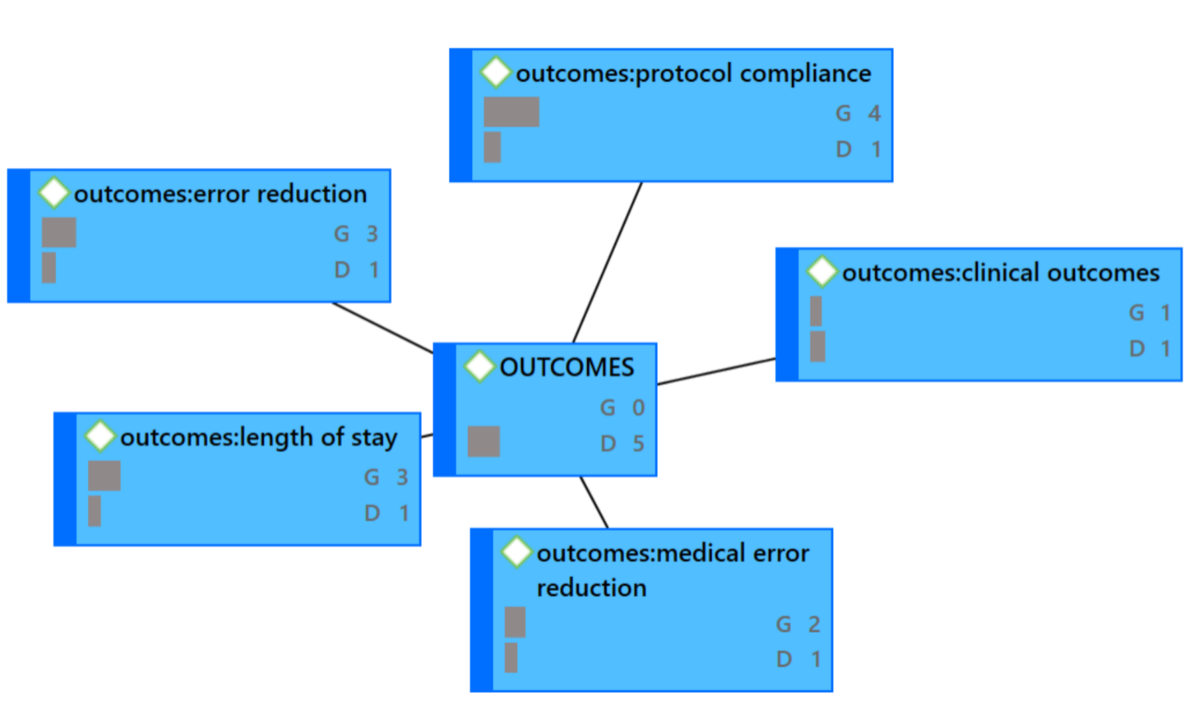
OUTCOMES Network Diagram (G=groundedness, D=density).

**Figure 6:**
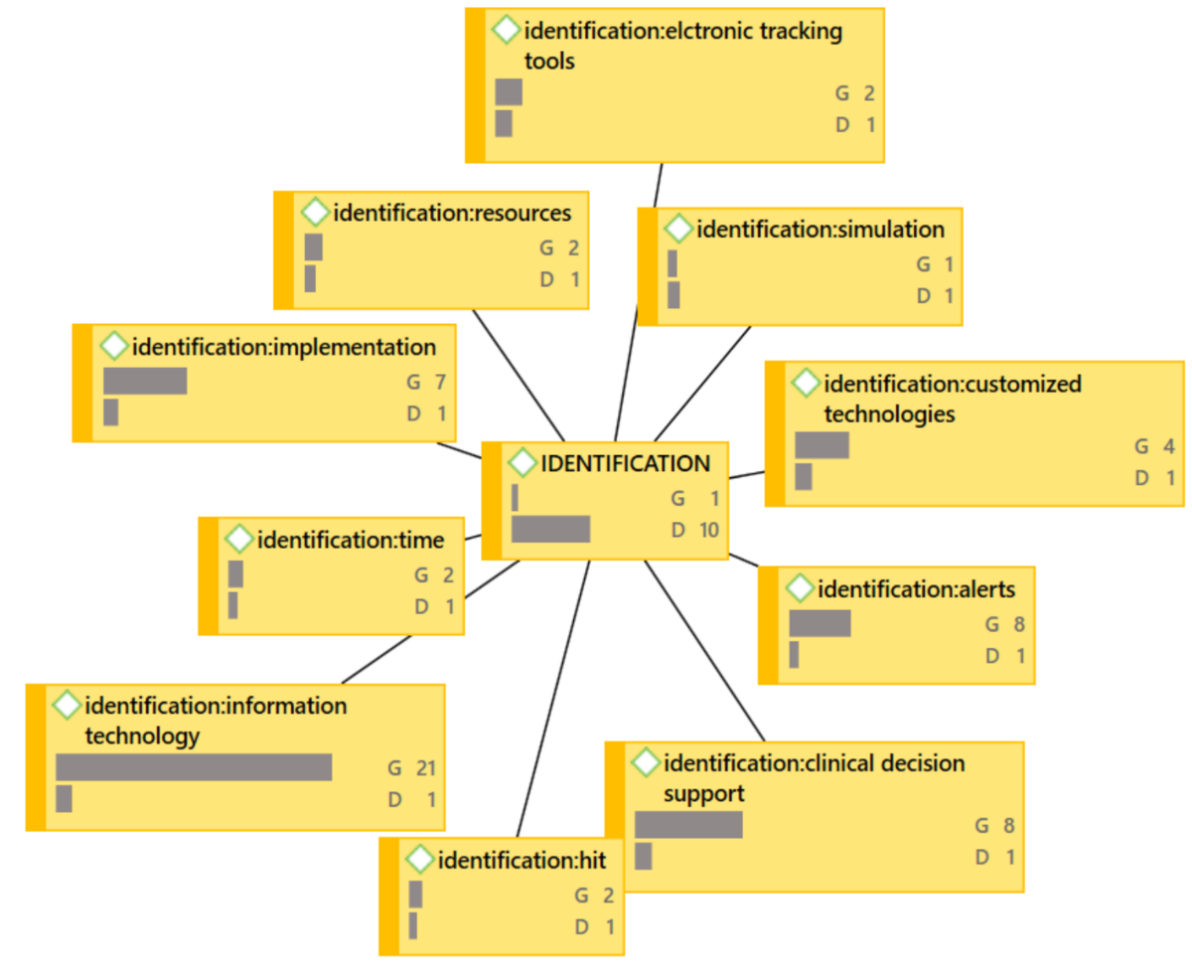
IDENTIFICATION Network Diagram (G=groundedness, D=density).

**Figure 7:**
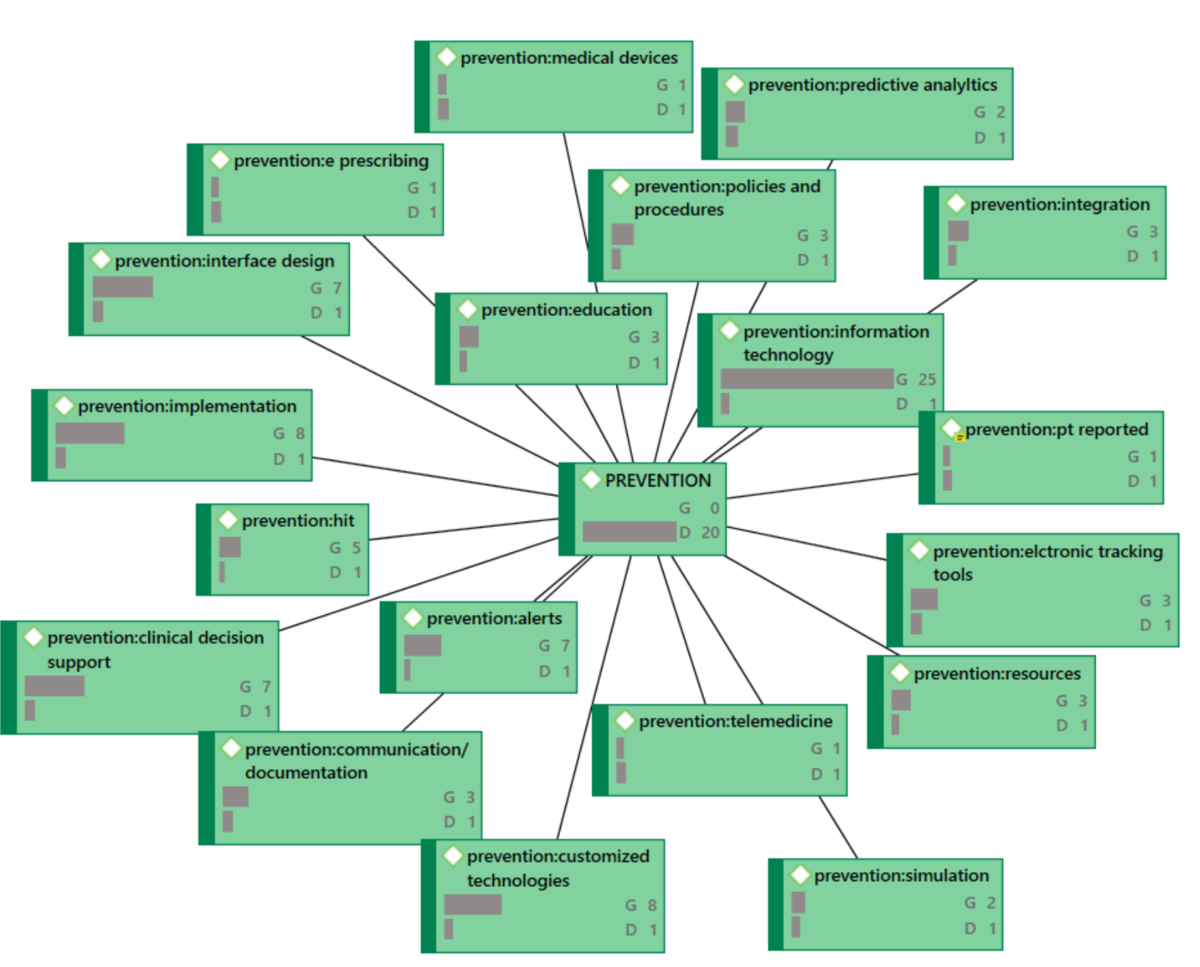
PREVENTION Network Diagram (G=groundedness, D=density).

